# Sex Differences in the Relationships between Forms of Peer Victimization and Reactive and Proactive Aggression in Schoolchildren

**DOI:** 10.3390/ijerph18105443

**Published:** 2021-05-19

**Authors:** Annis Lai-Chu Fung

**Affiliations:** Department of Social and Behavioural Sciences, City University of Hong Kong, Kowloon, Hong Kong, China; annis.fung@cityu.edu.hk

**Keywords:** sex difference, peer victimization, reactive aggression, proactive aggression, schoolchildren

## Abstract

The original study investigated sex differences in the relationships between multiple forms of peer victimization (physical victimization, verbal victimization, and social exclusion) and subtypes of aggression (reactive aggression and proactive aggression) in schoolchildren. A self-report questionnaire assessing levels of peer victimization and aggression was administered to 3790 schoolchildren (1916 males and 1874 females) aged 11 to 17 (*M* = 13.19; *SD* = 1.17) from 10 middle schools in Hong Kong. The pure effect of each subtype of aggression were evaluated by statistically controlling for another subtype of aggression in analyses. Furthermore, participants were classified as non-aggressors, reactive aggressors, proactive aggressors, and reactive–proactive aggressors to investigate their differences in specific forms of peer victimization. Data were analyzed by hierarchical linear regression and ANOVA. The results showed: (1) Sex significantly moderated the relationship between specific forms of peer victimization and subtypes of aggression; (2) In males, reactive aggression was positively predicted by verbal victimization; proactive aggression was positively predicted by physical victimization and social exclusion, and negatively predicted by verbal victimization; (3) In females, reactive aggression was positively predicted by physical victimization and social exclusion; proactive aggression was negatively predicted by social exclusion; and (4) Reactive–proactive aggressors reported more physical victimization than other types of aggressors. The findings have significant implications for distinctive functions of reactive and proactive aggression and the need to develop differentiated interventions for male and female schoolchildren.

## 1. Introduction

Peer victimization has been found to contribute to externalizing behavior such as aggression and delinquency [1], which may lead to further rejection and victimization by peers [2]. These consequences appear to be differentially associated with boys and girls. Particularly, a study revealed that involvement in delinquency is uniquely related to indirect forms of victimization (e.g., verbal victimization and social exclusion) in girls, although it increases the risk for direct forms of victimization (e.g., physical victimization) in both boys and girls [3]. On the other hand, it has been reported that proactive aggression increases with age in boys, while no sex difference has been reported for reactive aggression [4]. Such findings suggest that sex may moderate the relationship among different forms of peer victimization and subtypes of aggression. Yet, no study has examined sex differences in peer victimization (physical victimization, verbal victimization, and social exclusion) in relation to aggression subtypes (reactive aggression and proactive aggression) in schoolchildren.

Numerous studies have examined general aggression and general peer victimization but not the subtypes of aggression and forms of victimization [5,6,7,8,9,10]. Although the distinction between reactive and proactive aggression has been established for over three decades [11], few studies have investigated how reactive and proactive aggression in schoolchildren are related to peer victimization. Some findings have revealed a positive relationship between reactive aggression and peer victimization in schoolchildren [12,13,14,15], whereas the findings for proactive aggression are mixed [2].

Peer victimization refers to the experience of being targeted by peers with aggressive behavior that is intended to cause harm and is repeated over time, including physical, verbal, and social victimization [16]. Law and Fung [17] found that reactive aggressors, proactive aggressors, and reactive–proactive aggressors were all associated with higher levels of peer victimization (physical victimization, verbal victimization, social manipulation, and attacks on property) than were non-aggressors. However, a recent study [2] reported that although reactive aggression was positively related to all four forms of peer victimization, proactive aggression was positively linked with physical victimization, social manipulation, and attacks on property but was not significantly related to verbal victimization. This reveals inconsistency in the findings regarding the relationship between different forms of peer victimization to reactive and proactive aggression.

Moreover, a previous study [18] found that reactive aggression positively predicted future victimization for both boys and girls, whereas proactive aggression negatively predicted victimization only in boys. However, that study focused on general victimization, not multiple forms of peer victimization. Thus, this study fills a gap in the literature by examining the possible interaction effects of sex in the relationships between different forms of peer victimization and reactive and proactive aggression in schoolchildren.

### 1.1. Peer Victimization in Early Adolescence

Peer victimization is generally defined as a combination of: (i) physical victimization that is intended to cause pain or injury (e.g., kicking, punching, and beating); (ii) verbal victimization, such as name calling, swearing, and teasing about one’s appearance, race, or religious beliefs; (iii) social exclusion, which comprises rejecting, isolating, and corrupting behavior that is intended to cause the person to be ignored or turned against by their friends; and (iv) attacks on property, such as taking possessions without permission or deliberately damaging property [16,19,20].

Peer victimization is a common and salient social stressor throughout development, particularly during early adolescence when children transition to the upper grades in a different school environment and place more emphasis on peer relationships and social status [5,9,14]. As young adolescents experience such transitional changes, they may view aggression less negatively than younger children and become more willing to affiliate with aggressive peers, leading to increased aggression during early adolescence [9]. In the United States, 29.9% of a representative sample of over 15,000 students in grades 6 to 10 reported moderate or frequent engagement in bullying and peer victimization at school [8]. The developmental trajectory of peer victimization is generally expected to peak in the early middle school years [21,22].

### 1.2. Associations between Peer Victimization and Reactive–Proactive Aggression

Longitudinal studies have shown that peer victimization is prospectively linked with externalizing problems (including aggressive behavior) in children and adolescents, and children who externalize problems are more likely to behave in ways that irritate peers and provoke attacks [1]. This may in turn intensify their aggressive behavior to defend against such attacks, namely reactive aggression, leading to a vicious cycle of peer victimization that maintains their role as a victim [1,14].

Over the past three decades, reactive aggression has been distinguished from proactive aggression based on their distinctive functions and underlying purposes of actions [23]. Reactive aggression refers to an impulsive angry strike that functions as a defensive or retaliatory response to a perceived threat or provocation, whereas proactive aggression is described as deliberate coercive behavior that functions as an instrumental means to obtain desired goals or rewards [24,25]. The subtypes of aggression appear to be mediated by differences in social information processing (SIP), which affect how children process and interpret social cues and arrive at a decision that is socially competent (non-aggressive) or incompetent (aggressive; see Lemerise et al. [26]). Reactive aggression is associated with dysfunctional encoding and interpretation of social and affective cues in relation to the context (e.g., anger cues in ambiguous provocations elicit a hostile attributional bias), whereas proactive aggression is linked with positive outcome expectancies related to goal achievement and self-efficacy, which reinforce the intentional aggressive behavior [26].

Studies have consistently shown a specific relationship between peer victimization and reactive aggression among elementary and middle school children [15]. Peer victimization significantly predicted increased levels of reactive aggression across school years, while reactive aggression predicted more self-reported and teacher-reported peer victimization [14,15]. Especially among boys, those who experienced peer victimization tended to become more vulnerable to the influence of aggressive peers and displayed higher levels of reactive aggression over time [15]. Throughout development, repeated victimization by peers may lead children to develop a tendency to use reactive aggression to defend themselves or retaliate against perceived threats or provocations from peers [15]. In addition, they may become increasingly likely to pick up negative cues and attribute hostility to peers’ intentions, leading them to respond with impulsive anger or revengeful aggressive behavior [27].

Reactive aggression has also been found to predict hostile attribution bias and more frequent peer victimization across school years, even after controlling for prior levels of proactive aggression and peer victimization [14,28]. Specifically, in a recent study [29] that evaluated the links between physical and relational victimization and reactive and proactive aggression in young children based on teacher reports and observations, relational victimization among young children was positively predicted by reactive relational aggression, but negatively predicted by proactive relational aggression. Thus, children who exhibit reactive aggression appear to be more prone to peer victimization, and social exclusion in particular [12,14].

However, findings on the relationship between proactive aggression and peer victimization are inconsistent. A study that examined the specific forms of peer victimization showed that reactive aggressors reported more verbal victimization, whereas proactive aggressors reported more attacks on property [17]. Another study suggested a negative relationship between proactive aggression and victimization in boys but not in girls [18]. This leads to the question of whether sex moderates the levels of reactive and proactive aggression associated with different forms of peer victimization.

### 1.3. Potential Sex Differences in Peer Victimization and Aggression

Traditionally, males are more frequently involved in peer victimization than females, particularly physical aggression, but not relational aggression [22]. Compared with aggressive females, aggressive males are less likely to display physical aggression toward females [30], possibly due to the traditional norm that males “are expected to act as gentlemen to females, even when provoked” [31] (p. 474). Hence, males are more likely to be involved in physical victimization (e.g., beaten up, slapped, and pushed) and verbal victimization (e.g., derogatory statements about race/religion) that are intended to inflict physical and emotional pain. Females are more likely to experience verbal victimization (e.g., taunting and sexual comments) and social exclusion that is intended to harm the child’s reputation and social status [8,32,33].

In terms of aggression, a study conducted with a sample of over 5000 schoolchildren aged 11 to 15 found that boys showed more proactive aggression than girls as age increased, whereas no sex difference was found for reactive aggression [4]. However, in relation to peer victimization, only reactive aggression appears to be associated with a higher risk for peer victimization in boys at school, and it is unknown whether this association applies to different forms of victimization [14]. Currently, there is insufficient evidence to conclude how the different forms of peer victimization are related to reactive and proactive aggression in male and female schoolchildren.

### 1.4. The Present Study

This study fills a gap in the literature by examining the possible interaction effects between sex and peer victimization (physical victimization, verbal victimization, and social exclusion) based on the levels of reactive and proactive aggression in schoolchildren. Participants were classified as non-aggressors, reactive aggressors, proactive aggressors, or reactive–proactive aggressors, based on their levels of reactive and proactive aggression, to illustrate how specific forms of peer victimization may vary among ordinary children and those who exhibit different levels and subtypes of aggressive behavior at school.

Regarding sex difference in aggression subtypes, it was hypothesized that (i) males exhibit higher levels of proactive aggression than females. As for sex difference in peer victimization, it was expected that: (ii) males experience significantly more physical victimization than females; whereas (iii) females experience significantly more social exclusion than males. Furthermore, different forms of peer victimization differentially predict participants’ levels of reactive and proactive aggression, specifically that: (iv) physical victimization positively predicts proactive aggression; and (v) verbal victimization and social exclusion positively predict reactive aggression. In addition, (vi) sex and forms of peer victimization have significant interaction effects on the levels of reactive and proactive aggression in schoolchildren. With regard to subtypes of aggressors: (vii) reactive–proactive aggressors report the highest levels of all forms of peer victimization among all subtypes of aggressors; whereas (viii) reactive aggressors report significantly more verbal victimization and social exclusion than proactive aggressors and non-aggressors.

## 2. Materials and Methods

### 2.1. Participants

A questionnaire was completed by 3790 children (1916 males and 1874 females) aged 11 to 17 (*M* = 13.19; *SD* = 1.17) who were enrolled in grades 7 to 9 at 10 middle schools in Hong Kong. All participants were local Hong Kong Chinese.

### 2.2. Procedure

Ethical approval was obtained from the research committee of the university. The author openly invited all middle schools in Hong Kong (approximately 500) to participate in a study on school bullying, resulting in positive responses from 71 schools. Ten schools were selected by random number for participation. All students in grades 7 to 9 were invited to complete a questionnaire. Written parental consent was obtained. Both the children and their parents were informed that the study aimed to better understand the behavior and needs of children in Hong Kong and that the data collected would be kept anonymous and used for research purposes only.

### 2.3. Measures

The questionnaire consisted of the Reactive−Proactive Aggression Questionnaire (RPQ; [34]), the Peer Victimization Questionnaire (PVQ; [35]), and demographic questions such as age and sex.

#### 2.3.1. RPQ

The RPQ [34] is a self-report measure of aggressive behavior, consisting of 23 items rated on a 3-point Likert scale (0 = never; 1 = sometimes; and 2 = often), with 11 items for reactive aggression (e.g., “angry when frustrated”) and 12 items for proactive aggression (e.g., “use physical force to get others to do what you want”). Ratings were summed to yield subscale scores for reactive and proactive aggression. The Chinese version of the RPQ [4] was adopted. Cronbach’s alpha was 0.80 for reactive aggression and 0.84 for proactive aggression.

#### 2.3.2. PVQ

The PVQ [35] is a self-report measure of children’s peer victimization. Respondents were asked to report how often they experienced situations in the previous 3 months using a 5-point Likert scale (1 = never and 5 = always), with six items for physical victimization (e.g., “broke or destroyed my things”), eight items for verbal victimization (e.g., “said things to put me down”), and seven items for social exclusion (e.g., “refused to do things with me”). Higher scores represent more peer victimization. The PVQ was back-translated into Chinese for this study. The results from confirmatory factor analysis (CFA) suggested that the three-factor model of the Chinese PVQ was applicable to children in Hong Kong.

The 21 items of the PVQ were subjected to CFA using Mplus version 7.4 (Muthén & Muthén, Los Angeles, CA, USA). As the items were not normally distributed, the robust maximum likelihood estimation in Mplus, maximum likelihood parameter estimates with standard errors and a mean-adjusted chi-square test statistic that are robust to non-normality (MLM), was used. The results showed that the three-factor model had a better fit to the data, SRMR = 0.042, CFI = 0.91, TLI = 0.90, RMSEA = 0.046 (0.044, 0.048), ACI = 156,531.80, χ^2^ (186) = 1657.00, *p* < 0.001, than the one-factor model, SRMR = 0.049, CFI = 0.87, TLI = 0.85, RMSEA = 0.054 (0.052, 0.056), ACI = 158,071.11, χ^2^ (189) = 2261.27, *p* < 0.001. Peer victimization was better differentiated as three subtypes rather than as a single construct. Table 1 presented the factor loadings, average variance extracted (AVE), and composite reliability of the model. Although the item, “called me names,” had a low factor loading (i.e., <0.50), it was one of the common ways of verbal victimization and could not be replaced by other items. Thus, the author included it in the scale. The AVE of physical victimization and verbal victimization were <0.50, suggesting concerns for collinearity.

### 2.4. Data Analysis

Means of the scales (summed values of items divided by the number of items) were used for analyses.

Independent samples *t*-tests would be used to examine the sex differences in reactive aggression, proactive aggression, physical victimization, verbal victimization, and social exclusion (hypotheses (i), (ii), and (iii)).

Previous studies have reported high correlations between reactive and proactive aggression, ranging from 0.41 to 0.76 [11,34,36,37,38]. Therefore, proactive aggression would be controlled in the model predicting reactive aggression, while reactive aggression would be controlled in the model predicting proactive aggression so that the predictors of pure reactive aggression (independent of proactive aggression) and pure proactive aggression (independent of reactive aggression) could be evaluated.

Hierarchical linear regression analyses were used to test hypotheses (iv), (v), and (vi). In Step 1, reactive/proactive aggression was regressed on age and proactive/reactive aggression (as covariates), and sex (with male coded as the baseline), physical victimization, verbal victimization, and social exclusion. In Step 2, interaction terms (sex × physical victimization, sex × verbal victimization, and sex × social exclusion) were added to the model to assess the moderation effect by sex. To alleviate multicollinearity due to addition of the moderator and interaction terms, mean centering was performed on physical victimization, verbal victimization, and social exclusion. The regression models were evaluated by *R*^2^ and predictors were evaluated by standardized coefficients, β.

In addition, to examine the differences in peer victimization experiences among subtypes of aggressors and non-aggressors (i.e., reactive aggressors, proactive aggressors, reactive–proactive aggressors, and non-aggressors), participants were first classified based on their scores on the RPQ. Reactive–proactive aggressors scored z ≥ +1 for both reactive aggression and proactive aggression; reactive aggressors scored z ≥ +1 for reactive aggression only; proactive aggressors scored z ≥ +1 for proactive aggression only; and non-aggressors scored z < +1 for both reactive and proactive aggression, i.e., they did not meet the criteria for any of the aggressor subtypes. This classification criterion was adopted from previous studies [11,39,40]. Analysis of variance (ANOVA) was used to determine whether there were differences in peer victimization among the subtypes of aggressors and non-aggressors (hypotheses (vii) and (viii)).

## 3. Results

### 3.1. Sex Differences

The results of independent-samples *t*-tests indicated that males had significantly more proactive aggression, *t*(2938.16) = 10.08, *p* < 0.001; physical victimization, *t*(3178.37) = 13.07, *p* < 0.001; verbal victimization, *t*(3552.56) = 9.84, *p* < 0.001; and social exclusion, *t*(3644.88) = 7.68, *p* < 0.001, than females. The sex difference for reactive aggression was not significant, *t*(3721.72) = 0.34, *p* = 0.73. The means and standard deviations are presented in Table 1.

The dependent variables in the current sample were not normally distributed. Although the *t*-test is robust to departures from normality and homogeneity of variance, Mann–Whitney *U* tests were used for comparison. Consistent with the results from the *t*-tests, results from Mann–Whitney *U* tests revealed that males had significantly more proactive aggression, *U* = 1,525,626.00, *p* < 0.001; physical victimization, *U* = 1,390,267.50, *p* < 0.001; verbal victimization, *U* = 1,497,796.50, *p* < 0.001; and social exclusion, *U* = 1,545,587.50, *p* < 0.001, than females. The sex difference for reactive aggression was not significant, *U* = 1,746,114.50, *p* = 0.14.

### 3.2. Correlations

The correlation matrix is presented in Table 2. The Spearman’s correlations suggested that both reactive and proactive aggression were significantly and positively correlated with physical victimization, verbal victimization, and social exclusion, *ps* < 0.05.

### 3.3. Hierarchical Linear Regression

As both predictors and criteria were not normally distributed, a robust estimator in Mplus version 7.4 (Muthén & Muthén, Los Angeles, CA, USA), MLM, was used.

#### 3.3.1. Reactive Aggression

In Step 1, sex, age, proactive aggression, physical victimization, verbal victimization, and social exclusion significantly predicted reactive aggression: *R*^2^ = 0.315, *p* < 0.001. The addition of the interaction terms in Step 2 slightly increased the amount of variance explained. The final regression model accounted for 31.8% of the variance. Sex, age, proactive aggression, physical victimization, verbal victimization, and sex × physical victimization were significant predictors of reactive aggression, whereas social exclusion, sex × verbal victimization, and sex × social exclusion were not significant. In other words, after controlling for age and proactive aggression, a higher level of reactive aggression was predicted by more verbal victimization but less physical victimization in males, whereas it was predicted by more physical victimization in females. Furthermore, contrary to the results from the *t*-tests, females had more reactive aggression than males, after controlling for age and proactive aggression. Details of the final regression model are presented in Table 3. All VIF values were less than 10, indicating no signs of multicollinearity. Regarding effect sizes, proactive aggression (covariate) and verbal victimization had a medium effect, while the rest of the significant predictors only had a small effect.

#### 3.3.2. Proactive Aggression

In Step 1, the predictors significantly predicted proactive aggression: *R*^2^ = 0.312, *p* < 0.001. Moreover, the addition of the interaction terms in Step 2 slightly increased the variance explained. The final regression model accounted for 31.5% of the variance. In the final regression model, sex, reactive aggression, physical victimization, verbal victimization, social exclusion, and sex × social exclusion were significant predictors of proactive aggression, whereas age, sex × physical victimization, and sex × verbal victimization were not significant. In other words, after controlling for age and reactive aggression, a higher level of proactive aggression was predicted by more physical victimization and social exclusion, but less verbal victimization in males, while in females it was predicted by less social exclusion only. Details of the final regression model are presented in Table 4. As the VIF values were all less than 10, there were no signs of multicollinearity. Regarding effect sizes, all significant predictors only had a small effect.

### 3.4. Classification of Aggressors

The mean of reactive aggression in the sample was 4.50 (*SD* = 3.46), and the mean of proactive aggression was 0.81 (*SD* = 2.01). Based on the classification criteria, 2972 were non-aggressors, 444 were reactive aggressors, 163 were proactive aggressors, and 211 were reactive–proactive aggressors.

### 3.5. ANOVA

The means and standard deviations of different forms of peer victimization are presented in Table 5 by type of aggressor. The ANOVA results suggest that among the different subtypes of aggressors, there were significant differences in physical victimization, *F*(3, 3786) = 164.78, *p* < 0.001; verbal victimization, *F*(3, 3786) = 167.62, *p* < 0.001; and social exclusion, *F*(3, 3786) = 117.48, *p* < 0.001. The results from Games–Howell post hoc tests revealed that non-aggressors had the lowest levels of physical victimization, verbal victimization, and social exclusion, compared with aggressors. Moreover, reactive–proactive aggressors had the highest levels of physical victimization, verbal victimization, and social exclusion among the subtypes of aggressors. Proactive aggressors had a higher level of physical victimization than reactive aggressors, but they did not differ regarding verbal victimization and social exclusion. Details of the post hoc tests are presented in Table 6.

## 4. Discussion

The results indicate significant sex differences in the multiple forms of peer victimization and subtypes of aggression. Males scored significantly higher than females on proactive aggression and all forms of peer victimization, whereas females were uniquely associated with a higher level of reactive aggression, after controlling for proactive aggression. In addition, although all forms of peer victimization significantly predicted reactive and proactive aggression, sex significantly moderated the specific relationships between physical victimization and reactive aggression and between social exclusion and proactive aggression. The findings have significant implications for the distinctive functions of reactive and proactive aggression and the need to develop different interventions for male and female schoolchildren.

First, the results reveal meaningful sex differences in reactive and proactive aggression among schoolchildren. As expected, males exhibit more proactive aggression than females, and females display more reactive aggression than males. This may be explained by the different emphasis placed on interpersonal relationships by boys and girls. Eder [41] suggests that self-esteem in boys is associated with achievement (e.g., athletics); whereas girls’ self-esteem is tied to interpersonal relationships (e.g., popularity among peers). Thus, girls may rely more on their own and peers’ emotional cues as an important source of social information, which biases their encoding and interpretation of cues [26]. In ambiguous situations, girls may become more likely to detect negative emotional cues and attribute hostility to others’ intent, which triggers reactively aggressive responses to defend their personal image and peer status [23,42]. However, as boys tend to prefer achievement over peer relationships, they may show higher motivation to use goal-directed proactive aggression to obtain desired goals despite the potential negative costs, such as victim suffering and damage to friendships [43,44].

Unexpectedly, different forms of peer victimization did not seem to predict significantly different levels of reactive and proactive aggression. Both reactive and proactive aggression were positively correlated with all forms of peer victimization. This highlights the significant role of sex in moderating this relationship, such that the predictive links between specific forms of peer victimization and aggression subtypes were only evident when the sex of the children was considered. Nevertheless, when children were classified into subtypes of aggressor, proactive aggressors reported significantly more physical victimization than reactive aggressors and non-aggressors, whereas reactive–proactive aggressors scored the highest on all forms of peer victimization. This implies a unique association between proactive aggression and physical victimization. The moderation effects of sex on this association are discussed below.

Physical victimization predicted higher pure proactive aggression and lower pure reactive aggression in males but higher pure reactive aggression in females. This suggests that boys who experience physical victimization are more likely to use unprovoked aggressive behavior to obtain desired goals, whereas girls who experience physical victimization may be more likely to perceive hostility in others’ motives and display defensive or retaliatory aggressive behavior in response to provocations [24,45]. In line with the claim of Eder [41], boys’ self-esteem might be linked to personal achievement, especially athletics; hence, those who have been physically abused by peers may strive to enhance their self-esteem and power through aggressive acts towards peers [46]. Xu and Zhang [47] found proactive aggression to be strongly associated with positive outcome expectancies and self-efficacy beliefs in boys but not in girls. This supports the view that boys tend to perceive themselves as capable of performing aggressive acts and view aggression as an effective way to demonstrate their ability and power.

Similarly, social exclusion predicted higher pure proactive aggression in males, whereas in females, social exclusion predicted lower pure proactive aggression. This indicates that social exclusion may lead boys to display proactive aggression but not girls. A significant gender difference was found in social exclusion consistently in many previous studies that females are involved in social exclusion more than males [8,31,32,48,49]. If males experienced much social exclusion, they failed to adopt social skills to make friends and socialize with peer groups; therefore, they would prefer using proactively aggressive behaviors to control and manipulate others to show their dominance and power over their peers. On the contrary, when females experienced social exclusion, they internalized the peer victimization rather than being motivated to behave more proactively aggressively like males. In addition, adolescent boys were found to hold more positive views toward aggression than girls, perceiving aggression as a strategy to solve interpersonal conflicts and obtain peer status and power [46]. Based on the social learning theory [50], as the majority of warriors, fighters, and combatants in movies and electronic games are males, adolescent boys may learn from those figures, shifting from victims of social exclusion to a proactive aggressor role, as a leader in peer groups. Hence, boys who have been excluded or isolated by peers may resort to proactive aggression to gain social dominance over peers and prevent further social exclusion. Girls, in contrast, are more associated with internalizing problems, such as loneliness, anxiety, depression, and emotional dysregulation, that often intensify during the large social and physical transitions of adolescence [51]. As social exclusion behaviors are often intended to cause emotional pain in victims [16], girls who have experienced social exclusion may suffer from more severe internalizing problems, but not externalizing behaviors, such as reactive or proactive aggression. Furthermore, verbal victimization predicted higher pure reactive aggression and lower pure proactive aggression in males. However, it did not significantly predict pure reactive or proactive aggression in females. This reflects that boys who have been verbally assaulted display more reactive than proactive aggression. The literature suggests that verbal victimization does not merely take the form of conflicts or teasing (which could be friendly), but refers to words and acts that are intended to harm victims [16]. Children who have experienced verbal victimization by peers tend to show negative automatic thoughts and attributional styles, which mediate increases in depressive symptoms [52,53]. On this note, it could be inferred that verbal victimization contributes to more negative cognitions in children, resulting in biased interpretations of others’ words as malicious and the subsequent use of provoked aggression. However, as girls are already more prone to internalizing problems [51], negative changes in cognitive styles may be more evident in boys. Thus, boys who experience verbal victimization may demonstrate more reactive aggression than proactive aggression, which is driven by positive evaluations of self-efficacy and aggression outcomes.

As multiple forms of peer victimization have been shown to differentially predict reactive and proactive aggression in boys and girls, it can be concluded that sex plays a moderating role in the relationship between peer victimization and aggression. This pinpoints the need to develop differentiated intervention strategies for boys and girls, depending on their aggression subtypes and peer victimization experiences, to effectively reduce reactive and proactive aggression at school.

Nevertheless, this study has several limitations. First, although participants were asked to report peer victimization experiences retrospectively, causality between forms of peer victimization and reactive–proactive aggression remains unclear due to the cross-sectional nature of this study. Therefore, future studies could adopt a longitudinal design to investigate the causal relationship. Second, the results are based on self-report measures, which are prone to socially desirable responses. Aggressive behavior, particularly proactive aggression, is often viewed as detrimental to social harmony and challenging to authority figures and social rank in the Chinese cultural context [47]. Future studies could include parent, teacher, or peer ratings to provide a more comprehensive assessment of a child’s behavior.

## 5. Conclusions

This study provides substantial evidence for significant sex differences in the forms of peer victimization and aggression subtypes and interaction effects of sex and peer victimization on reactive and proactive aggression in schoolchildren. The findings have implications for the distinctive functions of reactive and proactive aggression in boys and girls undergoing the significant transitional changes of early adolescence. Intervention efforts should therefore include developing specific aggression reduction strategies for boys and girls based on their aggression subtypes and peer victimization experiences. However, more longitudinal research that includes informant ratings of children’s behavior is warranted to establish the causal relationship between specific forms of peer victimization and reactive–proactive aggression.

## Figures and Tables

**Table 1:** Details of the three-factor model of the PVQ.

Factor	AVE	CR	Item	Factor Loading
PV	0.41	0.81	Broke or destroyed my things	0.51
			Acted like they were going to beat me up or hurt me	0.69
			Beat me up or physically hurt me in some way	0.71
			Stole or took things from me	0.62
			Physically touched me in a way I did not want	0.64
			Threw things at me	0.67
VV	0.42	0.81	Called me names	0.36
			Said things to put me down	0.75
			Giggled or laughed at me	0.74
			Spread rumors about me	0.67
			Said things to put down people I like or care about	0.62
			Threatened to beat me up or hurt me	0.68
			Said things that offended me	0.75
SE	0.53	0.87	Left me out of things they were doing	0.69
			Gave me the “silent treatment” (did not talk to me on purpose)	0.74
			Refused to help me	0.74
			Did not invite me to parties, dances, social events, etc.	0.65
			Refused to do things with me	0.82
			Would not sit near me at lunch or in class	0.72
			Refused to share information or materials with me	0.79
			Tried to ditch or get rid of me	0.73

Note. AVE = Average Variance Extracted. CR = Composite Reliability. PV = Physical Victimization. PVQ = Peer Victimization Questionnaire. SE = Social Exclusion. VV = Verbal Victimization.

**Table 2:** Descriptive statistics of scales.

Scale	Male				Female				Total			
	*n* = 1916				*n* = 1874				*n* = 3790			
	*M*	*SD*	S	K	*M*	*SD*	S	K	*M*	*SD*	S	K
Reactive aggression	0.41	0.34	1.18	1.86	0.41	0.29	0.94	1.15	0.41	0.31	1.1	1.73
Proactive aggression	0.09	0.21	3.87	20.89	0.04	0.11	4.92	34.7	0.07	0.17	4.56	29.84
Physical victimization	1.18	0.53	2.39	7.08	0.99	0.32	3.27	14.14	1.09	0.45	2.85	10.44
Verbal victimization	1.39	0.65	1.71	2.98	1.21	0.49	2.26	6.52	1.3	0.59	1.98	4.45
Social exclusion	1.46	0.66	2.26	5.99	1.32	0.53	3.14	12.94	1.39	0.61	2.62	8.45

Note. K = Kurtosis. S = Skewness.

**Table 3:** Correlation matrix.

	1	2	3	4	5
1. Age	-	-	-	-	-
2. Reactive Aggression	−0.01	-	-	-	-
3. Proactive Aggression	0.03	0.41 **	-	-	-
4. Physical Victimization	0.07 **	0.31 **	0.33 **	-	-
5. Verbal Victimization	0.04 *	0.40 **	0.31 **	0.65 **	-
6. Social Exclusion	0.03	0.34 **	0.27 **	0.58 **	0.69 **

* *p* < 0.05. ** *p*< 0.01.

**Table 4:** Hierarchical regression models of sex and peer victimization on reactive aggression.

Step	*R* ^2^	*SE*	Factor	β	*SE*	*p*	VIF
1	0.315	0.02	Sex	0.10	0.01	<0.001	1.06
			Age	−0.03	0.01	0.02	1.01
			Proactive aggression	0.44	0.02	<0.001	1.18
			Physical victimization	−0.06	0.03	0.03	2.56
			Verbal victimization	0.30	0.03	<0.001	3.26
			Social exclusion	0.01	0.03	0.71	2.87
2	0.318	0.02	Sex	0.10	0.01	<0.001	1.07
			Age	−0.03	0.01	0.02	1.01
			Proactive aggression	0.44	0.02	<0.001	1.18
			Physical victimization	−0.09	0.03	0.001	4.20
			Verbal victimization	0.33	0.04	<0.001	5.49
			Social exclusion	−0.03	0.04	0.48	5.46
			Physical victimization × sex	0.06	0.03	0.04	2.98
			Verbal victimization × sex	−0.05	0.03	0.12	4.76
			Social exclusion × sex	0.05	0.03	0.13	4.46

Note. β = standardized coefficient. VIF = variance inflation factor.

**Table 5:** Hierarchical Regression Models of Sex and Peer Victimization on Proactive Aggression.

Step	*R* ^2^	*SE*	Factor	β	*SE*	*p*	VIF
1	0.312	0.02	Sex	−0.11	0.01	<0.001	1.05
			Age	0.02	0.01	0.08	1.01
			Reactive aggression	0.44	0.02	<0.001	1.18
			Physical victimization	0.25	0.03	<0.001	2.48
			Verbal victimization	−0.08	0.03	0.02	3.38
			Social exclusion	0.03	0.03	0.33	2.87
2	0.315	0.02	Sex	−0.24	0.03	<0.001	1.07
			Age	0.02	0.01	0.07	1.01
			Reactive aggression	0.13	0.01	<0.001	1.18
			Physical victimization	0.09	0.02	<0.001	4.12
			Verbal victimization	−0.02	0.01	0.04	5.65
			Social exclusion	0.02	0.01	0.04	5.46
			Physical victimization × sex	−0.01	0.03	0.73	2.99
			Verbal victimization × sex	0.01	0.02	0.46	4.76
			Social exclusion × sex	−0.03	0.02	<0.001	4.44

Note. β = standardized coefficient. VIF = variance inflation factor.

**Table 6:** Means and Standard Deviations of Forms of Peer Victimization by Type of Aggressor.

Type of Aggressor		Physical Victimization		Verbal Victimization		Social Exclusion	
	*n*	*M*	*SD*	*M*	*SD*	*M*	*SD*
Non-aggressors	2972	1.02 ^a^	0.37	1.20 ^a^	0.50	1.31 ^a^	0.51
Reactive aggressors	444	1.20 ^b^	0.54	1.58 ^b^	0.72	1.60 ^b^	0.81
Proactive aggressors	163	1.38 ^c^	0.61	1.57 ^b^	0.65	1.64 ^b^	0.71
Reactive–proactive aggressors	211	1.59 ^d^	0.66	1.90 ^c^	0.75	1.95 ^c^	0.82

Note. Means with differing superscripts (a, b, c, & d) within columns were significantly different at the *p* < 0.05 based on Games–Howell post hoc tests.

## Data Availability

The author confirms that the data supporting the findings of the study are available within this article.
